# Diagnostic Utility of Fluorine-18 Fluorodeoxyglucose Positron Emission Tomography/Computed Tomography in Infectious Diseases: A Retrospective Study

**DOI:** 10.7759/cureus.77991

**Published:** 2025-01-26

**Authors:** Tania García-Zaragoza, Francisco Jover-Diaz, Jorge Peris-Garcia, Elisabet Delgado-Sánchez, José Verdú-Rico, Blanca Lumbreras

**Affiliations:** 1 Infectious Diseases, University Clinical Hospital of San Juan de Alicante, Alicante, ESP; 2 Internal Medicine, Miguel Hernández University, Alicante, ESP; 3 Internal Medicine, Miguel Hernandez University, Alicante, ESP; 4 Nuclear Medicine, University Clinical Hospital of San Juan de Alicante, Alicante, ESP; 5 Public Health, History of Science, and Gynecology, Biomedical Research Network Consortium (CIBER) of Epidemiology and Public Health, Biomedical Research Center Network for Epidemiology and Public Health (CIBERESP), Madrid, ESP; 6 Public Health Sciences, Miguel Hernández University, Alicante, ESP

**Keywords:** 18f-fdg pet/ct, bacteremia, diagnostic confirmation, fever of unknown origin, infections, usefulness

## Abstract

Aim: This study aims to evaluate the utility of fluorine-18 fluorodeoxyglucose positron emission tomography/computed tomography (18F-FDG PET/CT) test for various infectious indications, analyze its diagnostic value, and assess its correlation with suspected diagnoses. Additionally, secondary endpoints include evaluating the test's usefulness in clinical management and identifying predictors of PET/CT positivity.

Methods: A retrospective descriptive observational study was conducted on in-hospital patients who underwent 18F-FDG PET/CT for suspected infections.

Results: A total of 89 patients were reviewed. The predominant symptoms were fever (68.5%) and respiratory symptoms (38.2%). The most common prior complementary tests were microbiological (89.9%) and imaging (94.4%). Indications for PET/CT included fever of unknown origin (27%), focal infections (20.2%), bacteremia (34.8%), and immunocompromised patients (18%). Diagnostic confirmation was achieved in 60.7% of cases. The test's usefulness (confirmation and unexpected findings) was 76.4%. Only 9.5% of patients without an initial diagnosis after PET/CT received a confirmatory diagnosis later. The subgroup analysis revealed that fever of unknown origin was associated with a lower probability of confirmation and usefulness, whereas focal infection was linked to a higher likelihood of diagnostic confirmation. However, these associations did not persist in multivariate analysis due to the limited sample size.

Conclusion: Our study aims to optimize the diagnostic profitability of the 18F-FDG PET/CT test for specific infectious disease indications by analyzing previous literature. In our experience, the PET/CT test demonstrated a high percentage of diagnostic confirmation for patients with focal infections. In contrast, its diagnostic yield was significantly lower for prolonged fever of unknown origin.

## Introduction

"Fever of unknown origin" (FUO) was first defined in 1961 by Petersdorf and Beeson [1] as a temperature of 101°F (38.3°C) or higher persisting for more than three weeks, without an established diagnosis after an intensive one-week investigation in the hospital. In 1991, Durack and Street [2] revised this definition, and it was recently updated by Surana et al [3].

In recent years, the diagnostic utility of 18F-FDG PET/CT in infectious processes has been investigated. It has proven to be a promising technique for detecting and extending infectious foci in various clinical conditions [4-15]. Additionally, it has shown usefulness in the therapeutic management of infections. Several studies have evaluated its diagnostic yield in infections, reporting rates between 26-92% [16-24]. Regarding sensitivity and specificity, 18F-FDG PET/CT has shown satisfactory results, with sensitivity between 70-93% and specificity between 35-89.8% [5,12,17,25,26]. The positive predictive value (PPV) and negative predictive value (NPV) of 18F-FDG PET/CT in infections have also been investigated, providing information on the probability that a positive or negative test result is accurate. The mean PPV and NPV in the reviewed studies were around 76% and 65%, respectively [5,12,17,25-26].

The advantages and limitations of the test must also be assessed. Advantages include accurate detection of the source of infection and guidance to an appropriate site for biopsy or other specific diagnostic tests [6,8-10,19-21,27]. It evaluates the patient globally [8,16], identifies infections in early stages [4,5,7,9,10,28], and allows the response to treatment to be evaluated, helping to adjust it and assess the effectiveness of antibiotics [4,5,8,9,16,20-24]. Limitations include reduced sensitivity in certain clinical processes, such as neutropenia [15], and affected specificity in low-prevalence infections [8,15]. The interpretation of results may be complex due to the presence of non-specific inflammation or neoplasms, making it difficult to differentiate between infection and other conditions, as well as interference from some drugs (antibiotics, immunosuppressants) [7-8,16]. Additionally, there is limited accessibility to testing in some centres [13,27].

Recently, it has been shown that 18F-FDG PET/CT may be more useful in certain types of infections, such as *S. aureus* bacteremias, as it can help identify the extent of the infection and its complications, and assist in therapeutic management [18-24,28-29]. Furthermore, due to the difficulty in distinguishing between neoplasms, infections, or inflammation with 18F-FDG PET/CT, new specific radiotracers are being studied for each condition [30].

These findings support the introduction of 18F-FDG PET/CT in diagnostic infection algorithms, as it can aid in the final diagnosis and management of patients with suspected infections [7,10,12,16-17]. However, in clinical practice, its use in infectious diseases is often restricted to difficult cases, particularly after an unsuccessful initial diagnostic study that may include imaging tests such as CT or MRI [6-9,13,16-17].

The primary endpoint of this study was to demonstrate the confirmation (correlation with suspected diagnoses) and usefulness (diagnostic value added) of the 18F-FDG PET/CT test in patients with various infectious pathologies such as (a) fever without a source or FUO in immunocompetent patients; (b) focal infection (with or without fever) in immunocompetent patients, such as central nervous system infections (abscesses, meningoencephalitis), COVID-19, osteoarticular infections (abscesses, osteomyelitis, spondylodiscitis), or prosthetic infections and (c) Bacteremias in immunocompetent patients (disseminated infection, prosthetic material, or endocarditis) and immunocompromised patients., e.g. neutropenic patients, HIV-infected individuals, or onco-hematological patients.

The secondary endpoint was to (a) assess the usefulness of PET/CT in the clinical management of patients and its predictors by comparing patient characteristics in positive and negative PET/CT cases and (b) compare blood test characteristics of positive PET/CT cases with those of negative PET/CT cases.

## Materials and methods

Study design

A retrospective descriptive observational study was conducted at the University Clinical Hospital of San Juan de Alicante, covering a population of 237,467 inhabitants with 394 hospitalisation beds, over six years and four months (2017-2023). In-hospital patients who underwent 18F-FDG PET/CT for suspected infections were evaluated. Data were retrospectively retrieved from hospital databases. Patients under 18, pregnant women, and those with active oncological or chronic inflammatory diseases or flare-ups were excluded.

Variables

Qualitative variables were described as numbers and percentages, while quantitative variables were expressed as medians and interquartile ranges due to their non-parametric nature. Qualitative variables were compared using Pearson's chi-square test or Fisher's exact test, while quantitative variables were compared using the Kruskal-Wallis test. A p-value <0.05 was considered statistically significant. Univariate analysis was performed on the main variables and factors associated with PET/CT positivity, followed by multivariate analysis using binary logistic regression for variables that were statistically significant or close to the threshold. Statistical analysis was performed using IBM SPSS software Version 22.0 (IBM Corp., Armonk, NY).

Ethical considerations

This protocol was designed following good clinical practices, the Helsinki Declaration, and other relevant Spanish legislation (Law 41/2002 on patient autonomy and Law 14/2007 on biomedical research). The study was approved by the institutional review board of the San Juan University Hospital of Alicante (approval no. 23/068). Due to the study's retrospective nature, the requirement for informed consent was waived as part of routine clinical practice.

## Results

A total of 101 patients were reviewed, but only 89 patients were included as they fulfilled the inclusion criteria. Table 1 describes the baseline characteristics of the sample (n=89) and by subgroups. The majority were men (n=64; 71.9%). The median age of participants was 65 years (51-79). The median hospital stay was 15 days (8-21). Cardiovascular comorbidities were the most prevalent: arterial hypertension (43.8%) and dyslipidaemia (38.2%). A total of 61 patients (68.5%) presented with fever (>101°F). Most cases (76.4%; n=68) presented with focal symptoms such as respiratory (38.2%), gastrointestinal (26.5%), urinary (20.6%), neurological (16.2%; n=11), or musculoskeletal symptoms (10.3%).

In total, 60 patients (67.4%) presented with systemic symptoms such as poor general condition (30%), asthenia (28.3%), low-grade fever, or weight loss (both 23.3%). SUVmax values were measured in 64 patients with a median of 9.1 (6.4-13.4). Some features were significantly more present in subgroups: implanted medical devices and hypertension was more frequent in patients with bacteremia, and pain and arterial hypertension were more common in patients with focal infection. Leukocytosis was significantly higher in those who had bacteremia. The results of PET/CT indications and their diagnostic utility are presented in Table 2. The most frequent indication for PET/CT was bacteremia (34.8%; n=31) to study its origin or complications, followed by FUO (27%; n=24), focal infection (20.2%; n=18), and immunosuppressed patients, including those with HIV infection (18%; n=16).

**Table 1:** Description of the characteristics of the patients included in the study (n=89). * Pearson's Chi-square test or Fisher's exact test; ^†^Kruskal–Wallis test. FUO: fever of unknown origin; IQR: interquartile range; IBD: inflammatory bowel disease; CRP: C-reactive protein; SUVmax: standardized uptake value maximum; Tª: temperature

Variables	Values; n=89	FUO (n=24)	Focal infection (n=18)	Bacteraemia (n=31)	Immunocompromised (n=16)	p-value
Sociodemographic /clinical characteristics						
Male	64 (71.9)	17 (70.8)	12 (66.7)	21 (67.7)	14 (87.5)	0.484*
Female	25 (28.1)	7 (29.2)	6 (33.3)	10 (32.3)	2 (12.5)	
Age (median IQR)	65 (51-79)	66 (47-77)	65 (57-79)	76 (62-83)	51 (46-62)	0.004†
Travel	4 (4.5)	1 (4.2)	1 (5.6)	0	2 (12.5)	0.272*
Previous episode	32 (36)	11 (45.8)	8 (44.4)	10 (32.3)	3 (18.8)	0.281*
Implanted medical devices	8 (9)	0	2 (11.1)	6 (19.4)	0	0.044*
Duration fever (days) (median IQR*)	2 (0-14)	15 (5-21)	1 (0-4)	1 (0-7)	1 (0-7)	0.002*
Length of stay	15 (8-21)	11 (7-16)	17 (11-28)	19 (13-28)	11 (5-17)	0.013*
Symptoms						
Systemic	60 (67.4)	14 (58.3)	11 (61.1)	24 (77.4)	11 (68.8)	0.448*
Focal	68 (76.4)	19 (79.2)	16 (88.9)	19 (61.3)	14 (87.5)	0.083*
Pain	34 (38.2)	6 (25)	12 (66.7)	10 (32.3)	6 (37.5)	0.038*
Fever	61 (68.5)	22 (91.7)	9 (50)	22 (71)	8 (50)	0.009*
Tª (median IQR)	38.1 (38-38.7)	38.3 (38-39)	38 (38-38.5)	38.2 (38-38.5)	38 (38-38.2)	0.341†
Complications	27 (30.3)	6 (25)	3 (16.7)	13 (41.9)	6 (31.3)	0.273*
Comorbities						
IBD	6 (6.7)	5 (20.8)	0	0	1 (6.3)	0.011*
Cancer	18 (20.2)	6 (25)	3 (16.7)	7 (22.6)	2 (12.5)	0.758*
Dyslipidaemia	34 (38.2)	8 (33.3)	11 (61.1)	12 (38.7)	3 (18.8)	0.078*
Diabetes mellitus	9 (10.1)	2 (8.3)	2 (11.1)	3 (9.7)	2 (12.5)	0.976*
Hypertension	39 (43.8)	8 (33.3)	11 (61.1)	18 (58.1)	2 (12.5)	0.007*
Other tests						
Microbiology	80 (89.9)	19 (79.2)	17 (94.4)	31 (100)	13 (81.3)	0.041*
Imaging	84 (94.4)	20 (83.3)	18 (100)	30 (96.8)	16 (100)	0.048*
Laboratory diagnosis	44 (49.4)	14 (58.3)	9 (50)	11 (35.5)	10 (62.5)	0.234*
CRP* (median. IQR*)	12.1 (5.5-19.4)	12.6 (6-21.1)	13.6 (9.1-19)	12 (5.9-19.6)	5.6 (2.1-14.6)	0.571†
Leukocytes (median. IQR*)	11800 (7300-14650)	11250 (7450-17150)	12155 (10400-17700)	12400 (9100-14400)	6850 (4750-10880)	0.044†
SUV*max (median/IQR*); n=64	9.1 (6.4-13.4)	7.3 (6.3-11.1)	10.4 (6.6-13.7)	7.1 (5.3-10.9)	9.6 (9-15.9)	0.088†
SUV*max (median/IQR*); n=64	9.1 (6.4-13.4)	7.3 (6.3-11.1)	10.4 (6.6-13.7)	7.1 (5.3-10.9)	9.6 (9-15.9)	0.088†

The results of PET/CT indications and their diagnostic utility are presented in Table 2. The most frequent indication for PET/CT was bacteremia (34.8%; n=31) to study its origin or complications, followed by FUO (27%; n=24), focal infection (20.2%; n=18), and immunosuppressed patients, including those with HIV infection (18%; n=16).

**Table 2:** Results of PET/CT and diagnostic indications (n=89). PET/CT: positron emission tomography/computed tomography; FUO: fever of unknown origin.

Variables	FUO; n=24 (27%)	Values; n(%)
Suspected diagnosis	Focal infection	18 (20.2%)
	Bacteraemia	31 (34.8%)
	Immunocompromised	16 (18%)
Diagnostic confirmation with PET/CT	Yes	54 (60.7%)
	No	35 (39.3%)
Diagnostic utility of PET/CT	Yes	68 (76.4%)
	No	21 (23.6%)
Diagnosis made by other means after a PET/CT scan		10 (11.2%)

In nine patients (9.5%), a definitive diagnosis was reached by another complementary test (biopsy or microbiological) after PET/CT was performed, regardless of whether PET/CT had been confirmatory or useful. A variety of diagnoses were finally confirmed, including visceral leishmaniasis, ganglionar tuberculosis, lung cancer, IgG4 disease, and systemic vasculitis. In only two cases, a negative PET/CT was performed, and a diagnosis was later established (Epstein-Barr virus mononucleosis and Adult Still's disease).

A univariate analysis (Table 3) found a statistically significant association for both diagnostic confirmation (60.7%; p=0.006) and the usefulness of the technique (76.4%; p=0.023) in all patients. Comparing both PET/CT subgroups, we observed that FUO patients had a lower probability of PET/CT confirmation (p=0.007) and a lower probability of usefulness (p=0.003). In contrast, patients with focal infection had a significantly higher probability of diagnostic confirmation (p=0.028). In our experience, PET/CT was less confirmatory and useful in FUO patients, as 52.4% of them (n=11) had normal results.

**Table 3:** Diagnostic confirmation and utility of PET/CT (n=89). ^*^Pearson's Chi-square test or Fisher's exact test. PET/CT: positron emission tomography/computed tomography; FUO: fever of unknown origin

Variables	n	Diagnostic confirmation; n(%)	p-value*	Utility; n(%)	p-value*
FUO	24	9 (37.5)	0.007	13 (54.2)	0.003
Focal infection	18	15 (83.3)	0.028	16 (88.9)	0.163
Bacteraemia	31	17 (54.8)	0.410	25 (80.6)	0.491
Immunocompromised	16	13 (81.3)	0.063	14 (87.5)	0.248
TOTAL (n)	89	54 (60.7)	0.006	68 (76.4)	0.023

Table 4 describes the univariate analysis of predictive factors and diagnostic confirmation. Patients with confirmed medical device infections were significantly detected by PET/CT (p=0.017). Fever was not significantly associated with a higher degree of diagnostic confirmation; however, a high percentage of patients without fever obtained diagnostic confirmation on PET/CT (p=0.019). Previous imaging tests were significantly associated with a higher degree of diagnostic confirmation (p=0.004). Although it did not reach statistical significance (p=0.071), the SUVmax value in PET/CT was higher in patients with diagnostic confirmation compared to those without.

**Table 4:** Description of the characteristics of the patients included in the study depending on whether the diagnosis is confirmed (n=89). *Pearson's chi-square test or Fisher's exact test. ^†^Kruskal–Wallis test. IQR: interquartile range; IBD: inflammatory bowel disease; CRP: C-reactive protein; SUVmax: standardized uptake value maximum.

Variables	n (%)	Diagnostic confirmation		p-value
		NO	YES	
Sociodemographic characteristics				
Sex				0.573^*^
Male	64 (71.9)	24 (68.6)	40 (74.1)	
Female	25 (28.1)	11 (31.4)	14 (25.9)	
Age (median. IQR range)	65 (51-79)	70 (50-83)	65 (54-76)	0.808^†^
Background				
Travel	4 (4.5)	1 (2.9)	3 (5.6)	0.548^*^
Previous episode	32 (36)	15 (42.9)	17 (31.5)	0.275^*^
Implanted medical devices	8 (9)	0	8 (14.8)	0.017^*^
Surgery	10 (11.2)	5 (14.3)	5 (9.3)	0.463^*^
Fever (days) (median. IQR)	2 (0-14)	4 (1-14)	2 (0-14)	0.545^†^
Length of stay	15 (8-21)	14 (8-19)	15 (8-22)	0.130^†^
Symptoms/signs				
Systemic	60 (67.4)	25 (71.4)	35 (64.8)	0.516^*^
Focal	68 (76.4)	25 (71.4)	43 (79.6)	0.373^*^
Swelling	6 (6.7)	1 (2.9)	5 (9.3)	0.239^*^
Heat	2 (2.2)	1 (2.9)	1 (1.9)	0.755^*^
Blush	2 (2.2)	0	2 (3.7)	0.250^*^
Pain	34 (38.2)	10 (28.6)	24 (44.4)	0.132^*^
Fever	61 (68.5)	29 (82.9)	32 (59.3)	0.019^*^
Temperature (median. RIQ)	38.1 (38-38.7)	38.2 (38-38.9)	38 (38-38.5)	0.477^†^
Complications	27 (30.3)	11 (31.4)	16 (29.6)	0.857^*^
Comorbidity				
IBD	6 (6.7)	3 (8.6)	3 (5.6)	0.579^*^
Cancer	18 (20.2)	8 (22.9)	10 (18.5)	0.619^*^
Dyslipidaemia	34 (38.2)	12 (34.3)	22 (40.7)	0.540^*^
Diabetes mellitus	9 (10.1)	5 (14.3)	4 (7.4)	0.293^*^
Hypertension	39 (43.8)	17 (48.6)	22 (40.7)	0.467^*^
Other tests				
Microbiological	80 (89.9)	31 (88.6)	49 (90.7)	0.740^*^
Imaging	84 (94.4)	30 (85.7)	54 (100)	0.004^*^
Laboratory confirmation	44 (49.4)	19 (54.3)	25 (46.3)	0.461^*^
CPR (median. IQR)	12.1 (5.5-19.4)	11.1 (5.5-19.3)	12.3 (6.5-19.5)	0.711^†^
Leukocytes (median. IQR)	11800 (7300-14650)	11800 (7800-18800)	11700 (6500-13900)	0.053^†^
SUVmax (median/IQR) n=64	9.1 (6.4-13.4)	5.50 (3.60-11.20)	15 (8-22)	0.071^†^
Total (n)	89	35	54	

In the multivariate analysis performed using binary logistic regression, there was a statistically significant absence of association between the different diagnostic suspicions and the analysis of the variables that reached statistical significance in the univariate analysis (existence of implanted medical devices, absence of fever, previous imaging tests).

## Discussion

Our retrospective study analyzes data from 89 patients who underwent PET/CT for bacteremia (34.8%), FUO (27%), focal infection (20.2%), and immunocompromised conditions (18%). The results of the main endpoints showed good PET/CT performance, with a high diagnostic confirmation rate (60.7%) and usefulness (76.4%). Only 9.5% of patients without a diagnosis after PET/CT obtained a confirmatory diagnosis later. FUO was associated with a lower probability of confirmation and usefulness, while focal infection was associated with a higher likelihood of confirmation.

Some case series and reviews [6,8-9, 15-16] have reported the usefulness of PET/CT in different infectious diseases. Most reviews [7, 13, 22, 25] and retrospective studies [5,11-12,14,17,26,29] analyze the usefulness of PET/CT in suspected FUO. Other studies [18,20-22, 24] evaluate its usefulness in bacteremic patients searching for the primary source of infection. Few prospective studies [10,19,28] have studied PET/CT for different indications. Weitzer et al. [4] described a small case series analysing the usefulness of PET/CT in different indications. We aimed to provide a broader analysis of PET/CT diagnostic utility in this infectious context, analysing various indications concerning the confirmation and usefulness of this technique.

The sample size of these studies, including ours (n=89), is a main limitation, as only three series have a sample size above 300 [5,26,29]. Among the baseline characteristics, it should be noted that bacteremic patients were more frequently hypertensive (p=0.007) due to greater cardiovascular comorbidity. In FUO patients, a significantly lower number of imaging tests were performed before PET/CT (p=0.048) compared to other indications. This may represent the clinician's confidence in the diagnostic utility of PET/CT for this indication. However, its diagnostic confirmation and usefulness degree were significantly lower, contrary to expectations. Thus, the indication of PET/CT in patients with FUO must be individualized, optimizing previous complementary tests.

We found an overall PET/CT diagnostic confirmation rate of 60.7% (p=0.006) and a utility rate of 76.4% (p=0.023). Considering that most patients had previous imaging techniques (94%) and microbiological diagnostic tests (90%), the performance of PET/CT significantly contributed to final diagnoses. In the literature, diagnostic yield ranges from 26 to 92% [5,7,12,15,17,24-26] for single diagnostic suspicions (e.g., FUO or bacteremias). A higher diagnostic utility in our series could be related to an established indication in our patients.

Analysis of confirmation and usefulness of PET/CT indications represents our main contribution to the literature. We found that FUO leads to the lowest diagnostic yield, both in confirmation and diagnostic utility, compared to what is proposed in the literature [5,7,12,15,17,24-26]. In our experience, PET/CT provides a significantly higher (83.3%; p=0.028) confirmation degree for focal infections. PET/CT increased diagnostic utility (15.7%) by detecting findings not suspected beyond the initial indication and contributed to unexpected diagnoses in fourteen patients.

Literature has scarcely described predictors of diagnostic confirmation [10,11,13]. Ghanem-Zoubi et al. [19] described a meaningful relationship between PCR values and the detection of an infectious source in bacteremic patients. Wang et al. [10] found that in FUO patients, the earlier PET/CT scan was performed (less than six days after admission) and higher CRP values (>9.5 mg/dl) improved the diagnostic accuracy of the test. Zhu et al. [11] confirmed this observation, as CRP >10 mg/dl was significantly associated with a higher diagnostic yield of PET/CT. However, this was not detected in our case series.

We found that implanted medical devices and previous imaging diagnostic tests were associated with greater diagnostic confirmation. However, fever was not associated with a greater diagnostic yield. Therefore, afebrile patients should not be ruled out as candidates for this imaging technique, although some patients may have already received antibiotic treatment at the time of the technique. Although SUVmax values were higher in cases of greater diagnostic confirmation, this association did not reach statistical significance. The limited sample size of our study may explain this fact.

Our study has some limitations or biases to be mentioned. As a single-centre study, generalization of the results to other populations or settings cannot be assumed. Limited sample size may impair the generalization of the results to a wider population. To overcome this limitation, it is our purpose to maintain prospective data collection and perform a sample size calculation to accurately assess the presence of predictive factors. Finally, its retrospective design could imply some bias in data collection. However, most data for analysis in our series (99.7%) were collected.

## Conclusions

In conclusion, in our experience, the PET/CT test has a high degree of diagnostic confirmation in patients with focal infections. Conversely, in FUO cases, the diagnostic yield was significantly lower and should be restricted to the final steps in diagnostics. Although some factors have been associated with diagnostic PET/CT in a multivariate study, this association was not detected. Establishing predefined indications for PET/CT could potentially optimize the diagnostic yield of the test.
